# RETRACTED: Jain et al. Lysosomes in Stem Cell Quiescence: A Potential Therapeutic Target in Acute Myeloid Leukemia. *Cancers* 2022, *14*, 1618

**DOI:** 10.3390/cancers16132317

**Published:** 2024-06-25

**Authors:** Vaibhav Jain, Swaroop Bose, Awadhesh K. Arya, Tasleem Arif

**Affiliations:** 1 Abramson Cancer Center, Department of Medicine, 421 Curie Blvd., Philadelphia, PA 19104, USA; vaibhav.jain@pennmedicine.upenn.edu; 2 Department of Dermatology, Mount Sinai Icahn School of Medicine, New York, NY 10029, USA; swaroop.bose@mssm.edu; 3 Department of Anesthesiology, Shock, Trauma and Anesthesiology Research Center, University of Maryland School of Medicine, Baltimore, MD 21201, USA; aarya@som.umaryland.edu; 4 Department of Cell, Developmental, and Regenerative Biology, Mount Sinai Icahn School of Medicine, New York, NY 10029, USA

The *Cancers* Editorial Office retracts the article entitled ‘Lysosomes in Stem Cell Quiescence: A Potential Therapeutic Target in Acute Myeloid Leukemia’ [1], cited above.

Following publication, concerns were raised to the Editorial Office regarding a potential overlap between this review article [1] and a previously published paper [2] with a different authorship group and without appropriate citation.

Adhering to our complaint’s procedure, an investigation was conducted by the *Cancers* Editorial Office and Editorial Board, which confirmed an overlap, which included figures and text, between this review article [1] and the prior publication mentioned above [2]. The authors fully cooperated with the investigation and the Editorial Office and Editorial Board, and the authors have decided to retract this article [1] as per MDPI’s retraction policy (https://www.mdpi.com/ethics#_bookmark30).

This retraction was approved by the Editor-in-Chief of the journal *Cancers*.

The authors agree to this retraction.
